# A virtual teaching clinic for virtual care during the COVID-19 pandemic

**DOI:** 10.1186/s40842-020-00108-1

**Published:** 2020-11-25

**Authors:** Xin He, Daniel Shelden, Andrew Kraftson, Tobias Else, Richard J. Auchus

**Affiliations:** 1 Department of Internal Medicine, Division of Metabolism, Endocrinology & Diabetes, University of Michigan, 1500 East Medical Center Drive, Ann Arbor, MI 48109 USA; 2 Department of Pharmacology, University of Michigan, 1150 West Medical Center Drive, Ann Arbor, MI 48109 USA; 3 Veterans Affairs Ann Arbor Healthcare System, 2215 Fuller Road, Ann Arbor, MI 48105 USA

**Keywords:** Telemedicine, Virtual care, Preceptor, Resident, Fellow

## Abstract

The COVID-19 pandemic has prompted the rapid transition of in-person outpatient care to telemedicine, and clinical training to remote learning. The endocrinology fellows at the University of Michigan maintain their own continuity-of-care clinics and rotate in the Ann Arbor Veterans Affairs (VA) Healthcare System. For these clinics, we sought to preserve patient staffing with expert attending physicians and continue the clinical training experience in a remote setting.

We have adapted the online conferencing platform, Zoom, to integrate learners into a virtual teaching clinic environment. By using the Zoom “breakout room” feature, fellows are able to match staffing attending physicians to different patient cases, according to attending physicians’ areas of specialty. Similar to the traditional teaching clinic environment, our remote staffing strategy has ensured that fellows continue to provide excellent patient care and fulfill educational aims across our University and VA facilities.

Outpatient clinics in other University of Michigan departments and other academic centers have inquired about or have begun utilizing our method. Even beyond COVID-19, our paradigm potentially provides a convenient virtual staffing platform to serve patient populations with geographic or transportation challenges. Following implementation, stakeholders can regularly evaluate the approach to continually improve both patient care and medical education.

## Background

The COVID-19 pandemic has brought profound challenges across all spheres of medicine, including medical education. Trainees typically acquire outpatient clinical knowledge through in-person care of patients with attending physician supervision. Outpatient care has transitioned to telemedicine visits, and training curricula have shifted to remote learning. With trainees and faculty members sheltering at home, we sought a solution that allows multiple providers to provide education and patient care from different locations.

## Transition to virtual teaching clinic

The endocrinology fellows at the University of Michigan maintain a continuity-of-care clinic, in which different attending physicians rotate every week to staff patients. Before the COVID-19 pandemic, four attending endocrinologists would be stationed in workrooms, and the eight fellows would staff each patient with an available attending physician, seeking also to match a faculty physician’s area of expertise to the given complaint. The endocrinology clinics at the Ann Arbor Veterans Affairs (VA) Healthcare System operate under a similar paradigm with trainees and attending endocrinologists. When outpatient clinics rapidly transitioned to telemedicine, we endeavored to create an efficient virtual environment, which preserved expert patient staffing and continued the educational experience in a remote setting.

Online conferencing platforms have become indispensable tools for remote work and group teaching. We adapted a Michigan Medicine secure Zoom (San Jose, CA) platform to integrate learners into a virtual teaching clinic environment. Zoom provides a “breakout room” feature, which allows the conference “host” (a Chief Fellow) to set up private communications among participants outside of the main session. The host assigns each conference participant to one breakout room; the participants can then switch between their single assigned breakout room and the main session, but cannot visit other breakout rooms. While it was simple to assign each attending physician a unique breakout room, it was challenging to create a virtual clinic that enabled fellows to rotate among faculty-specific breakout rooms.

To overcome this cumbersome inconvenience in the Zoom environment, all fellow participants are assigned “co-host” status. As co-hosts, each fellow has the ability to self-reassign to different breakout rooms, without involving the host. This scheme obviates the need for the meeting host to change room assignments during clinic, thus creating efficient and flexible virtual staffing rooms.

**Fig. 1:**
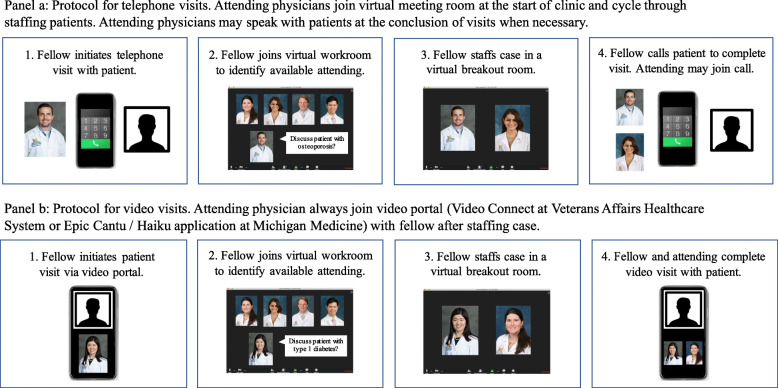
Panel **a** Protocol for telephone visits. Attending physicians join virtual meeting room at the start of clinic and cycle through staffing patients. Attending physicians may speak with patients at the conclusion of visits when necessary. Panel **b** Protocol for video visits. Attending physician always join video portal (Video Connect at Veterans Affairs Healthcare System or Epic Cantu / Haiku application at Michigan Medicine) with fellow after staffing case

At the start of clinic, all attending physicians congregate in the Zoom main session, while fellows begin video and telephone patient encounters. Each time a fellow is ready to staff a case, the fellow joins the main session, identifies the most appropriate attending physician available, and moves to the corresponding breakout room (Fig. 1). Telephone visits are completed when the fellow calls the patient back to discuss the final plan, with the option of the attending physician joining in a three-way telephone call. For video visits, the fellow and attending physician join the patient in a three-way video visit through the Epic electronic medical record platform or VA Video Connect platform – which are muted or discontinued during the actual staffing discussion. After discussing the case, the attending physician then returns to the main session to staff subsequent patients. This method allows fellows to staff patients with different attending physicians by moving freely amongst the respective breakout rooms.

When our fellows’ continuity-of-care clinics were converted to telemedicine with virtual staffing, the clinic appointment templates were not changed, and patient volume during the pandemic decreased slightly. In the month of April 2019, each fellow saw an average of 5.2 patients per four-hour half day, and each attending physician staffed an average of 7.8 patients. In comparison, in April 2020, fellows saw 4.5 patients, and attending physicians staffed 7.2 patients. While detailed time studies were not completed, our subjective sense is that decreased patient volume and absence of physical exams shortened staffing wait times and duration of patient visits.

## Conclusions

Our strategy has ensured that fellows continue to provide the best care to our patients in our University and VA facilities. Similar to the traditional teaching clinic environment, fellows seek the guidance of our experts across different areas, despite all providers and patients being physically miles apart. In addition to providing excellent patient care and fulfilling educational aims, this process also meets supervision requirements for billed services by the attending physicians. Furthermore, ad-hoc expert faculty can join the clinic for a specific patient as needed, simply by joining the Zoom platform. We use widely available technology that is easy to set up and maintains Health Insurance Portability and Accountability Act (HIPAA) compliance. Even beyond the COVID-19 pandemic, our paradigm provides a convenient virtual staffing platform to serve patient populations with geographic or transportation challenges, while easing the burden of provider commutes. Following implementation, stakeholders can regularly assess the platform to continually improve both medical care and education. For example, patients can be surveyed on their satisfaction with virtual care. Trainees can evaluate the quality of teaching received, and faculty can provide feedback on learner performance. Training programs can perform time studies to quantify the relative duration of patient history-taking, staffing discussions, and staffing wait times. Healthcare systems can monitor outcome metrics for patients receiving virtual versus in-person care. The virtual teaching clinic approach can be an iterative one, easily adapted to other training program clinics to maintain the mission of teaching hospitals and clinics.

## Data Availability

Not applicable.

## Abbreviations

VA: Veterans Affairs
HIPAA: Health Insurance Portability and Accountability Act

